# The Socioeconomic Factors and the Indigenous Component of Tuberculosis in Amazonas

**DOI:** 10.1371/journal.pone.0158574

**Published:** 2016-06-30

**Authors:** Daniel Barros de Castro, Rosemary Costa Pinto, Bernardino Cláudio de Albuquerque, Megumi Sadahiro, José Ueleres Braga

**Affiliations:** 1 Health Surveillance Foundation of Amazonas State, Manaus, Brazil; 2 Sergio Arouca National School of Public Health, FIOCRUZ, Rio de Janeiro, Brazil; 3 Institute of Social Medicine, Rio de Janeiro State University, Rio de Janeiro, Brazil; 4 PVS PECTI-SAÚDE/Research Foundation of the State of Amazonas, Manaus, Brazil; Public Health Agency of Barcelona, SPAIN

## Abstract

Despite the availability of tuberculosis prevention and control services throughout Amazonas, high rates of morbidity and mortality from tuberculosis remain in the region. Knowledge of the social determinants of tuberculosis in Amazonas is important for the establishment of public policies and the planning of effective preventive and control measures for the disease. To analyze the relationship of the spatial distribution of the incidence of tuberculosis in municipalities and regions of Amazonas to the socioeconomic factors and indigenous tuberculosis component, from 2007 to 2013. An ecological study was conducted based on secondary data from the epidemiological surveillance of tuberculosis. A linear regression model was used to analyze the relationship of the annual incidence of tuberculosis to the socioeconomic factors, performance indicators of health services, and indigenous tuberculosis component. The distribution of the incidence of tuberculosis in the municipalities of Amazonas was positively associated with the Gini index and the population attributable fraction of tuberculosis in the indigenous peoples, but negatively associated with the proportion of the poor and the unemployment rate. The spatial distribution of tuberculosis in the different regions of Amazonas was heterogeneous and closely related with the socioeconomic factors and indigenous component of tuberculosis.

## Introduction

Tuberculosis (TB) is considered a serious public health problem in Brazil, with about 70,000 new cases and 5,000 deaths registered annually [1]. Brazil is one of 22 countries that account for 80% of the TB cases worldwide and, in 2013, ranked 16th in the list of countries with the highest number of new cases [2].

The Brazilian state of Amazonas had the highest TB incidence rate in 2013, with 70.6 cases per 100,000 inhabitants. In the same year, the average incidence rate in the country was 35.4 cases per 100,000 residents. Moreover, Amazonas had the highest mortality rate in the northern region and the third-highest mortality rate among Brazilian states, with 3.5 deaths per 100,000 residents [1].

In recent decades, efforts have been made to broaden and improve the quality of disease management services offered in Amazonas through the decentralization of actions for basic health units [3]. However, the actions taken are based primarily on the diagnosis and directly observed treatment, according to the guidelines recommended by the Ministry of Health [4].

TB control strategies mainly focuses on cutting transmission through early case detection and effective treatment, although having presented a significant contribution to reducing the disease burden in several countries, have a limited impact [5]. The literature shows that living conditions, housing, work, income, education, and access to public services are barriers to health-care access and that knowledge about the distribution of the disease according to population characteristics allows for the development of intervention strategies that consider cultural, epidemiological, and operational characteristics.

Amazonas has an indigenous population of approximately 168,000, which represents 20% of Brazil's indigenous population [6]. These populations are known to be vulnerable to the occurrence of diseases due to unfavorable socioeconomic conditions and lack of health services [7]. Moreover, studies indicate that the Amazonian indigenous population present a possible immunological susceptibility to tuberculosis [8,9]. It is highlighted that the demographic census of 2010 indicated that the state of Amazonas has a high proportion of poor people, great inequality of income distribution, and an average human development index of 0.674 [10].

This study analyzed the relationship of the spatial distribution of the incidence of TB in the municipalities and regions of Amazonas to socioeconomic factors, the performance of health services, and the indigenous TB component.

## Materials and Methods

An ecological study was conducted based on secondary data from an epidemiological surveillance of TB, in which the units of analysis were municipalities of the state of Amazonas, Brazil.

Secondary data from TB epidemiological surveillance routine of Amazonas state were used and the data allowing identification of the individuals in the database were excluded by the technical staff of the Center for Information Systems of Health Surveillance Foundation for Health of Amazonas. Thus, none of the authors interacted with patients or had access to identifying information. There was authorization of database use by the board of Health Surveillance Foundation of Amazonas State and this study was approved by the Institutional Review Board of the State University of Amazonas (#1.418.145). The research was conducted under ethical conditions in accordance with the Helsinki declaration.

**Fig 1:**
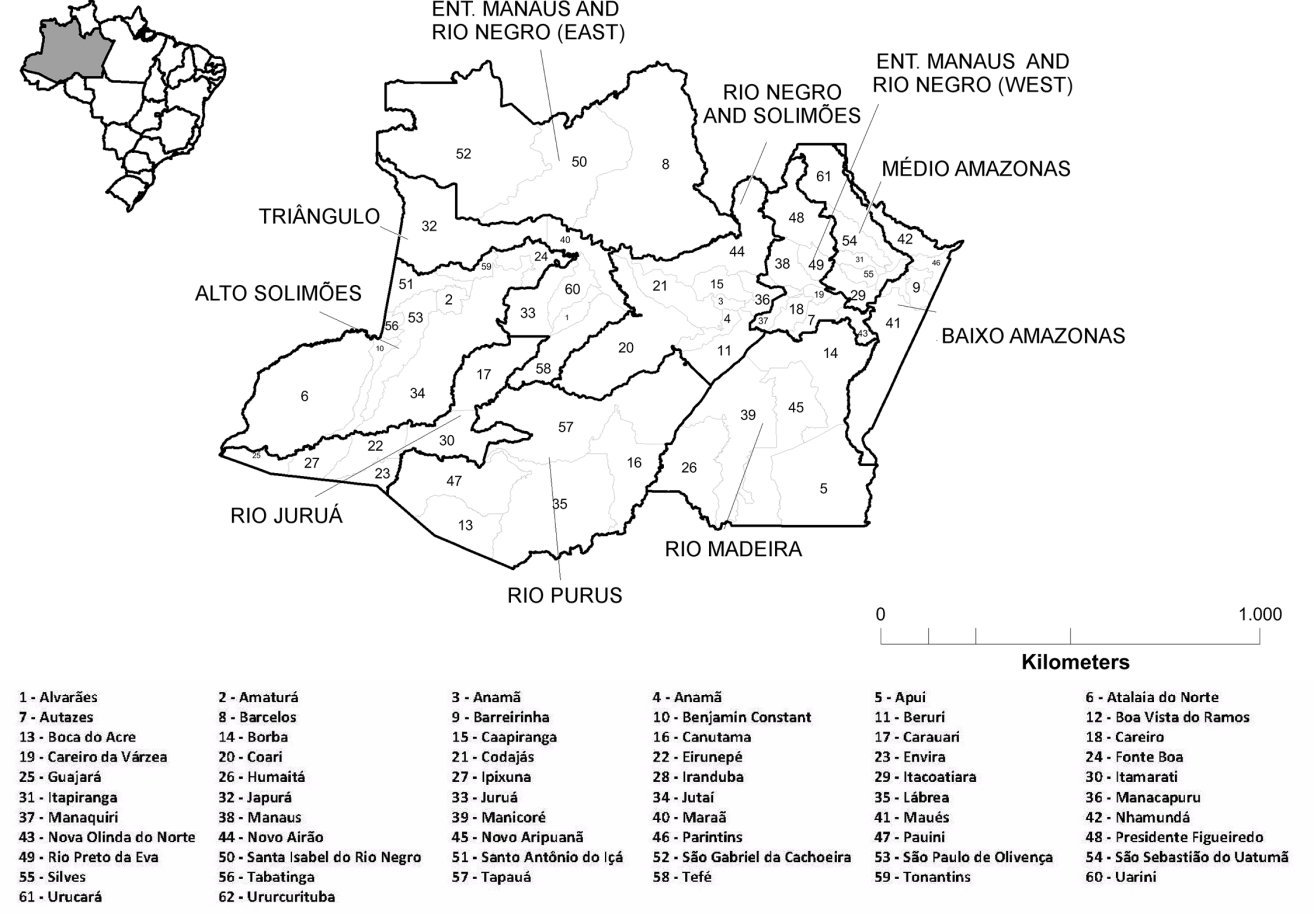
Municipalities and health regions of the state of Amazonas. Source: Brazilian Institute of Geography and Statistics (IBGE).

The state of Amazonas, located in the northern region of the country, has a total area of 1,559,161 sq km, and is made up of 62 municipalities and 9 health regions. The state has a population of 3,483,985, of whom 1,802,525 live in the capital, Manaus. The region was originally made up of two geographically separate areas, namely Entorno de Manaus and Rio Negro, and for the purpose of analysis in this study, these areas were disaggregated and called West and East. The former comprised the municipalities of São Gabriel da Cachoeira, Santa Isabel do Rio Negro, and Barcelos. The latter was made up of the other nine municipalities in this region (Fig 1).

Data on TB cases were extracted from the National Disease Notification System, which is provided by the Health Surveillance Foundation of the Secretariat of the State of Amazonas Health Department. All new TB cases in the resident population of Amazonas reported between January 1, 2007, and December 31, 2013, were selected, regardless of sex or age. The following records were excluded: duplicate records (n = 15); those that had been attributed a “change of diagnosis” (n = 239); and those where the “race/color” option was blank or had been filled in with “unknown” (n = 272).

TB cases were classified according to whether the subjects were indigenous or non-indigenous. Cases were classified as TB in indigenous subjects in records that had the field “ethnicity/color” filled in as “indigenous.” The cases classified as non-indigenous had “ethnicity/color” filled in variably as “white,” “black,” “yellow,” or “brown.” This information was obtained from patients’ responses when completing the case report form.

The municipality age- and sex-adjusted incidence rate was calculated from the individual data records of cases of tuberculosis. The cases were classified geographically according to the place of residence of the patient. We calculated the average age- and sex-adjusted incidence rate, and multiplied by 100,000.

The socioeconomic information used was the municipal human development index (MHDI), Gini index (income inequality of household heads), average household income per capita, proportion of the poor population (those with per capita income below half the minimum wage), proportion of poor children, and unemployment rate of the population aged 18 years or older. For these indicators, the data for the year 2010, provided by the United Nations Development Program, were used [11]. Considering that the detection of TB cases depends on the proper functioning of health services, a performance measurement of health services, called the performance index of the Unified Health System, was used, composed of access and effectiveness indicators for the year 2010, made available by the Ministry of Health [12].

To measure the indigenous TB component, the attributable fraction for TB occurring in the indigenous population was calculated from the incidence of TB in the general population in the state of Amazonas, based on TB incidence rates in indigenous and non-indigenous people. The proportion of outcomes (TB cases in Amazonas) assigned to cases of TB in indigenous peoples was calculated by using the population attributable fraction (PAF), a measurement widely applied in public health and TB research [13,14]. This measure is often used to assess the effect of eliminating the risk factor for certain outcomes, thus allowing the measurement of how much the incidence of TB in Amazonas can be reduced if all TB cases in the indigenous population were eliminated.

The PAF of TB relating to indigenous peoples was calculated as a function of the relative risk according to the following formula:

$$\text{PAF}=\frac{n_{11}}{n_{.1}}\times \frac{RR-1}{RR}$$

RR is the relative risk of becoming ill from tuberculosis among indigenous peoples in comparison with non-indigenous, n11 represents the number of indigenous people who became ill with tuberculosis, and n.1 represents the total number of individuals who became ill with tuberculosis.

The population living in municipalities and regions was obtained from censuses conducted by the Brazilian Institute of Geography and Statistics (IBGE) in 2000 and 2010. From these data, populations for the years in between were estimated by applying the linear interpolation technique [15]. The distinction between indigenous and non-indigenous populations in these censuses was made from the responses of the subjects when questioned about their color or race [6]. Digital cartographic meshes of municipalities and regions were obtained from the IBGE.

To study the relationship between socioeconomic factors and the incidence of TB in the municipalities of Amazonas, a multiple linear regression model was used. It was also appreciated collinearity and the interaction between the explanatory variables in the analysis that led to the final multiple regression model. For the final model, only those that presented a relationship with the outcome at a significance level of 0.10 were selected by using the backward stepwise technique. Thus, measures of association were calculated between the average annual incidence of TB and the socioeconomic factors, besides the PAF of TB of the indigenous people.

For the analysis, the statistical package STATA v.13 (StataCorp, College Station, Texas, USA) was used. For creating thematic maps, the Quantum Geographic Information Systems (QGIS) v.2.10.1-Pisa (OSGeo, Beaverton, OR, USA) application was used.

## Results

From 2007 to 2013, a total of 15,418 TB cases were reported in the state of Amazonas, wich correspond to an average annual incidence of 62 cases per 100,000 inhabitants. The highest incidence rate was found in the region where the state capital is located, in Entorno de Manaus and Rio Negro (East), with 74 cases per 100,000 inhabitants (Table 1).

**Table 1:** Average rate of incidence of TB in the general population, indigenous and non-indigenous populations, and the population attributable fraction of TB in the indigenous populations in the health regions and state of Amazonas from 2007 to 2013.

State/Region	Area (km^2^)	Total population^a^	Indigenous population^a^	Average annual TB cases (CI^b^)	Average annual age and sex-adjusted incidence rate per 100,000 inhabitants (CI^b^)	Average annual incidence rate in the non-indigenous population per 100,000 inhabitants (CI^b^)	Average annual incidence rate in the indigenous population per 100,000 inhabitants (CI^b^)	Indigenous TB population attributable fraction (%)
Amazonas	1,559,148	3,483,985	168,680	2,189 (2,034–2,344)	62.4 (59–65)	61.5 (58–64)	81.1 (68–93)	1.5
Alto Solimões	213,235	224,094	61,901	105 (94–115)	51.1 (47–55)	42.3 (37–46)	54.2 (41–66)	7.0
Baixo Amazonas	68,382	214,881	11,993	98 (79–116)	46.7 (38–54)	42.6 (33–51)	76.0 (43–108)	4.2
Entorno (East)^c^	70,756	2,037,985	14,222	1,578 (1,433–1,723)	74.6 (69–79)	76.9 (72–81)	99.9 (70–129)	0.2
Entorno (West)^d^	294,432	81,760	48,133	55 (38–73)	70.5 (47–93)	30.7 (25–36)	95.2 (55–135)	54.4
Médio Amazonas	58,424	149,130	671	70 (56–85)	47.2 (37–56)	46.4 (37–55)	62.8 (43–81)	0.2
Rio Juruá	93,205	117,043	4,756	35 (30–42)	36.1 (29–42)	19.1 (13–24)	303.2 (138–468)	37.5
Rio Madeira	221,081	165,663	10,402	60 (46–75)	38.6 (28–48)	32.0 (22–41)	112.1 (37–186)	14.0
Rio N. Solimões	157,223	252,027	2,485	108 (89–127)	45.8 (37–53)	42.9 (36–48)	44.3 (8–80)	0.0
Rio Purus	250,417	118,314	8,235	38 (31–45)	34.8 (26–43)	30.3 (21–39)	65.4 (46–84)	7.7
Triângulo	131,990	123,088	5,882	40 (34–45)	35.9 (30–41)	32.0 (26–37)	21.9 (4–38)	-1.5

^a^2010 census data.

^b^95% Confidence Interval.

^c^Entorno Manaus and Rio Negro (East).

^d^Entorno Manaus and Rio Negro (West).

In most regions of the state, the average annual incidence of TB in the indigenous population is higher than that in the non-indigenous population. An exception was observed only in the Triângulo region. However, in the Rio Juruá region, the rate of the indigenous population is 15 times higher than that of the non-indigenous population (Table 1).

The PAF of TB in the indigenous populations in the state of Amazonas during the study period was 1.5%. However, in Entorno de Manaus and Rio Negro (West), and Rio Juruá, the PAF of TB in the indigenous peoples was high at 54.4% and 37.5%, respectively (Table 1).

A negative association was found between the average rates of the annual incidence of TB and the poverty and unemployment rates. Moreover, a positive association was found between the TB incidence rate, Gini index, and PAF of TB in indigenous peoples. The coefficients indicate a greater association with poverty rate, followed by the unemployment rate, Gini index, and indigenous PAF, respectively (Table 2).

**Table 2:** Factors associated with the incidence of tuberculosis in municipalities of the state of Amazonas from 2007 to 2013.

Factor	Mean (sd)	Min	Max	Regression coefficient	P value	95% Confidence interval
Gini index	0.61(0.05)	0.52	0.80	110.7	0.037	6.88 to 214.63
Indigenous PAF	0.22(0.23)	0.01	0.92	31.3	0.026	3.97 to 58.73
Unemployment rate^a^	73.8(9.6)	33.5	88.6	−5.6	0.000	−2.06 to −0.96
Proportion of the poor^b^	7.6(3.3)	1.6	18.9	−2.8	0.008	−3.61 to −0.58

^a^Unemployment rate at 18 years of age.

^b^Proportion of the poor population.

**Fig 2:**
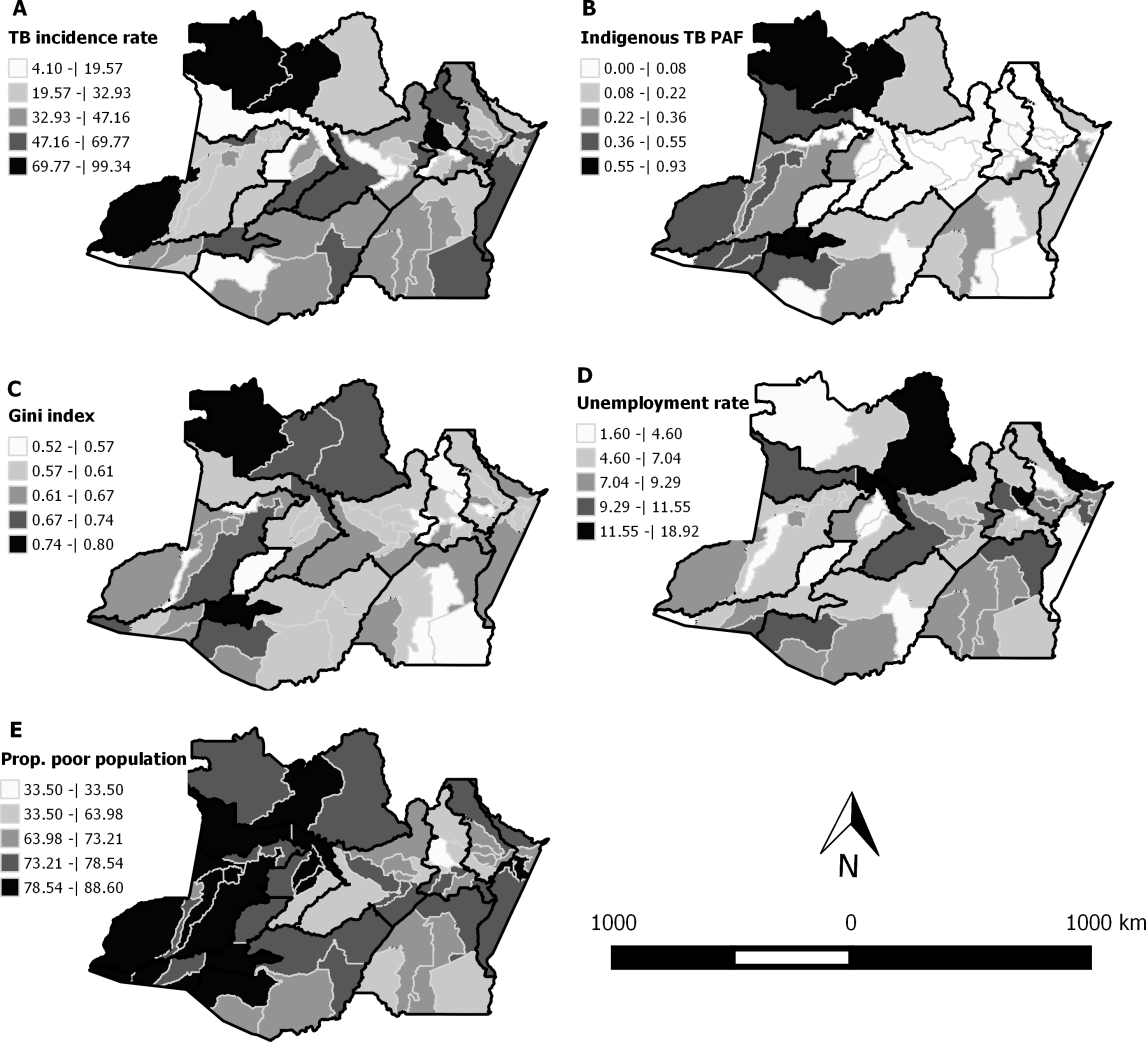
Spatial distribution of the TB incidence, socioeconomic factors, and the indigenous TB component in the state of Amazonas, from 2007 to 2013. (A) Age- and sex-adjusted TB incidence rate. (B) PAF of TB in indigenous peoples. (C) Gini index. (D) Unemployment rate at 18 years of age. (E) Proportion of the poor population.

Socioeconomic factors and the indigenous TB component partly explained the incidence of TB in the municipalities of the state of Amazonas. As shown in Fig 2, the municipalities with high TB incidence rates (Map A) and higher Gini index (Map B) and higher PAF of TB in indigenous populations (Map C) are located in the north of the state, especially in the Alto Rio Negro region and in the southwest, in the Rio Juruá region. Municipalities that have lower unemployment rates (Map D) and proportion of poor people (Map E) are located close to the capital, east of the state.

## Discussion

TB is distributed heterogeneously in cities and regions of Amazonas, and only two regions have a low incidence. Our main findings indicate that the indigenous situation plays a small part in explaining the incidence of TB in Amazonas during the study period. Moreover, they reveal the importance of TB in the indigenous peoples in two regions, namely Entorno de Manaus and Rio Negro (West), and Rio Juruá. Although TB in indigenous people accounts for 1.5% of the endemic, its PAF is a condition independently associated with the incidence of the endemic in the municipalities of Amazonas.

In Brazil, as in other countries with a high burden of TB, this endemic has mainly affected populations with poor living conditions and it is concentrated in large urban centers [16,17]. The spatial distribution of TB in the state of Amazonas that was characterized in this study reveals a pattern that can be explained by two different conditions: (i) higher incidence in densely populated areas such as in the capital, Manaus, and (ii) in populations with a large indigenous contingent as in the regions of Entorno de Manaus and Rio Negro (West), and Rio Juruá. As a large portion of the Amazonian population is of indigenous descent, a synergistic effect of these two factors is likely to occur in populations like those living in Manaus that had a large population growth in the last decade owing to migration, including indigenous people seeking better living conditions [18].

Indigenous peoples have long been known to be a population group vulnerable to TB and other major endemic diseases in the Amazon region [19–21]. However, whether this condition justifies or explains the high incidence rates recorded in state of Amazonas for at least 40 years is not sufficiently clear. The susceptibility of indigenous populations to TB led the Brazilian government to direct efforts to control the endemic through specific actions such as the creation of the service for special attention to indigenous peoples, which was named SUSA, and featured the great sanitarian Noel Nutels as a major figure in the history of Brazilian public health [22]. Currently, the National Tuberculosis Control Program recommends a set of priority actions for TB control, specific to indigenous areas of Brazil [4]. This study aimed to contribute to the understanding of whether indigenous TB is able to account for the magnitude of TB in the population of Amazonas, that is, how much of the endemic is due to the occurrence in patients of the indigenous population.

Our findings confirm the higher risk of illness from TB among indigenous populations than in non-indigenous ones. The small influence of the indigenous condition in the occurrence of TB in Amazonas indicates that other aspects such as poor living conditions and the huge population density present in large cities should be considered so that the behavior of this endemic disease can be understood. Moreover, this study indicates that TB in indigenous peoples greatly influences the incidence of TB in municipalities in regions such as Rio Juruá and Rio Madeira.

Extensive literature states that TB is a disease strongly determined by socioeconomic factors [23–25]. Economic inequality appears to have a major influence on the health status of the population [26]. In this study, economic inequality measured by the Gini index showed that more unequal populations have a higher risk of illness. Moreover, contrary to what was found in other studies, we observed an inverse relationship of the incidence of TB to the proportion of the poor population and the unemployment rate. It could be contradictory with the former finding, but we should highlight that in Brazil, poor people have limited access to effective health services to obtain diagnosis and treatment of TB [27]. This statement may explain the occurrence of cases in economically developed urban centers in the state of Amazonas. This idea is also supported by the relationship between the performance indicator of the UHS and these socio-economic indicators. In the study population health services performance was related to proportion of the poor population (correlation coefficient equal -0.55) and health services performance also was related to the unemployment rate (correlation coefficient equal -0.15), although this association was not maintained with statistical significance in the multiple regression model.

It is also noteworthy that even if the PAF of the indigenous condition contributes little to the magnitude of the endemic, it is a condition that is associated with the incidence of TB, regardless of economic inequality, the proportion of the poor population, and the unemployment rate.

The limitations of this study include the possibility of underreporting cases of TB, either because of problems related to coverage and access to services offered to the population and possible errors in classification and/or diagnosis of TB cases reported in Amazonas. Furthermore, the self-classification of the race (indigenous and non indigenous) can be considered a limitation of the study because some people would not self-declared as indigenous due to the stigma it could generate. On the other hand, others can declare themselves as indigenous because of the benefits offered by the Brazilian government [28]. Despite these limitations, the findings are useful for making a public health decision, as they indicate areas of priority for the development of actions aimed at the prevention and control of TB, with a focus on indigenous populations.

## Conclusions

The spatial distribution of TB in different regions of the state is heterogeneous, maintaining a close relationship with the socioeconomic conditions and the proportion of the indigenous population. The increased vulnerability of indigenous peoples to TB was noted, when compared with that of the non-indigenous population, although the burden of TB in Amazonas is only slightly influenced by the cases occurring in this population.
